# Early Onset of Cerebral Vasospasm and the Mediating Impact of Secondary Infarction on In-Hospital Mortality Following Aneurysmal Subarachnoid Hemorrhage

**DOI:** 10.3390/diagnostics16040551

**Published:** 2026-02-13

**Authors:** Gregor Peter, Lukas Meyer, Bogdana Tokareva, Gabriel Broocks, Matthias Bechstein, Vincent Geest, Christian Heitkamp, Felix Schlicht, Luca Meucci, Lasse Dührsen, Hanno S. Meyer, Helge Kniep, Maxim Bester, Jens Fiehler, Christian Thaler

**Affiliations:** 1 Department of Diagnostic and Interventional Neuroradiology, University Medical Center Hamburg-Eppendorf, Martinistraße 52, 20251 Hamburg, Germanyb.tokareva@uke.de (B.T.); gabriel.broocks@medicalschool-hamburg.de (G.B.); m.bechstein@uke.de (M.B.); v.geest@uke.de (V.G.);; 2 Department of Neuroradiology, HELIOS Medical Center, Campus of MSH Medical School Hamburg, 19055 Schwerin, Germany; 3 Department of Neurosurgery, University Medical Center Hamburg-Eppendorf, 20251 Hamburg, Germany

**Keywords:** aneurysmal subarachnoid hemorrhage, cerebral vasospasm, in-hospital mortality, mediation analysis, secondary infarction

## Abstract

**Background:** Cerebral vasospasm (CV) as a complication after aneurysmal subarachnoid hemorrhage (aSAH) is a major determinant of secondary vasospasm-associated ischemic infarction (SVS-I) and poor outcome. Data on the interplay among the onset of CV, SVS-I, and in-hospital mortality remain limited. **Methods:** We conducted a retrospective, single-center study including patients admitted with aSAH between January 2016 and May 2024 who developed treatment-relevant CV. The primary outcome was the rate of in-hospital mortality. The relationship between the onset of CV, demographics, imaging, and treatment data and the primary outcome was analyzed using logistic regression. A confounder-adjusted mediation analysis was performed to quantify the extent to which the effect of time to CV onset on in-hospital mortality was mediated by SVS-I. **Results:** A total of 165 patients with aSAH and treatment-relevant CV were included. The median age was 55 (IQR, 48–64), and 67.2% (111) were female. Of the included patients, 13.3% (22) died during hospitalization. In multivariable logistic regression analysis, earlier onset of treatment-relevant CV (adjusted odds ratio [aOR] 0.79; 95% CI, 0.66–0.95) and the occurrence of SVS-I (aOR 13.47; 95% CI, 2.78–65.3) were associated with the primary outcome. Mediation analysis indicated that SVS-I accounted for 28% of the effect of earlier onset of CV on in-hospital mortality. **Conclusions:** Twenty-eight percent of the effect of earlier onset of cerebral vasospasm on in-hospital mortality was mediated by secondary ischemic infarction. Targeting patients with early-onset vasospasm and the associated risk of infarction may reduce in-hospital mortality following aneurysmal subarachnoid hemorrhage.

## 1. Introduction

Cerebral vasospasm (CV) is a feared complication following acute aneurysmal subarachnoid hemorrhage (aSAH) [1,2,3,4]. The occurrence of CV is known to be associated with increased odds for poor outcomes [5]. CVs occur most often within the first 14 days following aSAH; however, the onset of CV varies among patients [6,7,8]. Early onset of CV often necessitates repetitive endovascular interventions (e.g., intra-arterial vasodilator administration or mechanical dilatation). It is a major risk factor for the development of secondary vasospasm-associated ischemic infarction (SVS-I) [9,10,11,12,13,14,15]. Thus, this subgroup of aSAH patients is at risk of very poor outcomes and in-hospital mortality [16,17]. Nevertheless, the interplay between individual patient characteristics and the severity of CV during the acute in-hospital phase, and their association with outcomes, remains unknown. In particular, reliable early risk stratification remains challenging, as clinical deterioration may be delayed and imaging or monitoring findings are not always concordant across patients. Therefore, identifying readily available predictors linked to early CV onset and subsequent SVS-I could help refine surveillance strategies and guide timely escalation of endovascular therapy.

The aim of this study was to analyze the relationship between the time to onset of CV, SVS-I, and other individual patient characteristics, as well as their impact on in-hospital mortality. We hypothesized that an early onset of CV is associated with ischemic infarction and increased rates of in-hospital mortality.

## 2. Materials and Methods

The study protocol was reviewed and approved by the local ethics committee of Hamburg, Germany (Chamber of Physicians, Hamburg), and was conducted in full accordance with the ethical principles outlined in the Declaration of Helsinki and its subsequent amendments. Given the retrospective nature of the study and the use of routinely collected clinical data, the requirement for written informed consent was waived by the ethics committee. All patient data were handled in compliance with applicable data protection regulations and institutional policies to ensure confidentiality and anonymity. The design, conduct, and reporting of this study adhered to the Strengthening the Reporting of Observational Studies in Epidemiology (STROBE) guidelines for cohort studies, ensuring transparent and comprehensive reporting of methods and results.

### 2.1. Study Population

All patients treated for aSAH at the University Medical Center Hamburg-Eppendorf between January 2016 and May 2024 were retrospectively identified and screened for eligibility. Clinical, radiological, and procedural data were extracted from electronic medical records and institutional imaging databases. Patients were included if they developed symptomatic CV following aSAH that was considered clinically relevant and required at least one endovascular intervention, defined as intra-arterial administration of vasodilatory agents and/or mechanical vessel dilatation by balloon angioplasty. The indication for endovascular treatment was based on the presence of new or worsening neurological deficits and/or perfusion abnormalities attributable to CV, in conjunction with angiographic confirmation.

**Figure 1:**
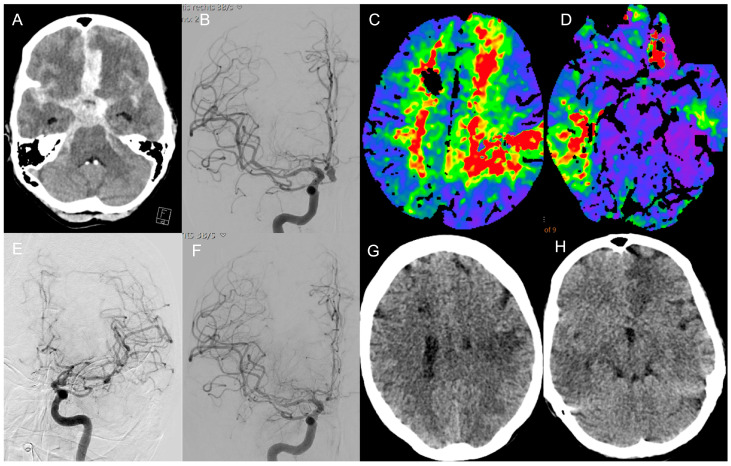
Representative case of early vasospasm. 56-year-old male patient with severe subarachnoid hemorrhage (**A**) due to an irregular ACoA aneurysm (**B**) treated with simple coiling. Three days later, the patient presented with elevated flow velocities in TCD. Subsequent CT perfusion demonstrated bilateral frontoparietal perfusion delay (**C**) and right temporal perfusion delay (**D**). Digital subtraction angiography (DSA) revealed moderate vasospasm of both the middle cerebral and anterior cerebral arteries (**E**,**F**). The patient underwent five sessions of intra-arterial spasmolysis in both territories of the internal carotid artery. The patient developed secondary infarctions in both MCA territories (**G**,**H**) as well as in the left ACA territory (**H**) and ultimately died during hospitalization. Note: focal parenchymal changes along the EVD catheter tract may be present and should not be interpreted as vasospasm-associated territorial ischemia.

**Figure 2:**
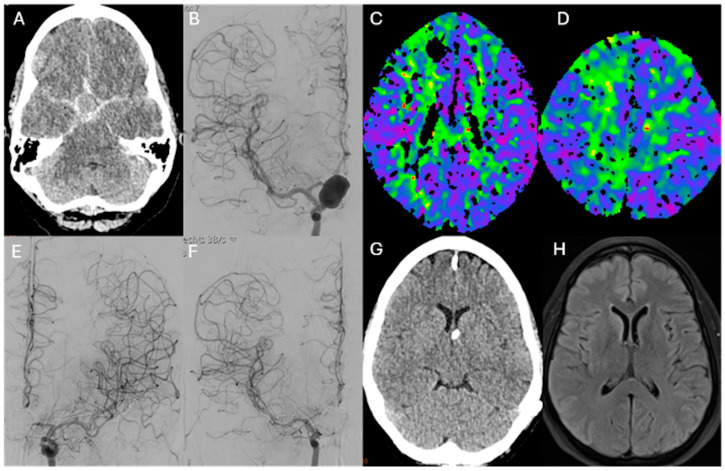
Representative case of late vasospasm. 52-year-old female patient with severe subarachnoid hemorrhage (**A**) due to a supraophthalmic ICA aneurysm (**B**) treated with stent-assisted coiling. Elevated flow velocities on transcranial Doppler (TCD) were detected on day 11, followed by CT perfusion demonstrating bilateral frontoparietal perfusion delay (**C**,**D**). Digital subtraction angiography (DSA) revealed moderate vasospasm of the bilateral middle cerebral and anterior cerebral arteries (**E**,**F**). The patient underwent three sessions of intra-arterial spasmolysis in both carotid territories. During the clinical course, no secondary infarctions developed (**G**,**H**). Note: focal parenchymal changes along the EVD catheter tract may be present and should not be interpreted as vasospasm-associated territorial ischemia.

Patients without symptomatic CV, those managed exclusively with conservative or medical therapy, or those with incomplete clinical or imaging data were excluded from further analysis. Figure 1 and Figure 2 illustrate representative angiographic and clinical cases of patients with early-onset cerebral vasospasm (Figure 1) and late-onset cerebral vasospasm (Figure 2), highlighting the interplay of the onset time of CV between infarct development and clinical outcome.

### 2.2. Management of Vasospasms

For vasospasm prevention, patients received nimodipine from the day of admission either orally (60 mg/4 h) or intravenously (1–2 mg/h). Maintaining a constant intracranial perfusion pressure of 60–70 mmHg was the main priority, with an alternative goal of keeping mean arterial pressure > 80 mmHg.

Transcranial Doppler (TCD) measurements were performed daily to detect relevant vasospasm. TCD ultrasound was performed by experienced physicians or technicians, and the mean velocity in the middle cerebral artery and anterior cerebral artery was measured. According to AWMF guidelines ^6^, TCD measurements were recorded as mean velocity (cm/s). Elevated TCD measurements in the middle cerebral artery were defined as either a mean velocity > 140 cm/s or a doubling within 24 h.

Patients who could not be clinically assessed, e.g., due to intubation, received daily TCD measurements and baseline computed tomography perfusion (CTP) within 24 h after aneurysm treatment. In case of normal TCD measurements, CT imaging with computed tomography angiography (CTA) and CTP was performed 4–6 days after admission to detect pronounced vessel narrowing or perfusion deficit. In some cases, this was repeated 10–14 days after admission.

If patients showed relevant vasospasm, they received endovascular treatment including intra-arterial administration of nimodipine (routinely injected in the ipsilateral internal carotid artery of the affected territory or vertebral artery in case of relevant vasospasm of the posterior circulation) or percutaneous transluminal angioplasty via balloon or temporary stent of the affected vessel segment. Treatment-relevant vasospasm was defined as angiographically confirmed vasospasm on DSA that led to endovascular rescue therapy (intra-arterial spasmolysis) in the presence of either (1) new or worsening neurological deficits attributable to vasospasm, and/or (2) a new territorially concordant perfusion deficit on CTP in the affected vascular territory. Endovascular treatment decisions were made in an interdisciplinary team involving neurosurgeons and neuroradiologists. The choice between pharmacological spasmolysis and mechanical dilatation was individualized based on vasospasm location, vessel caliber, angiographic severity, and clinical presentation. Intra-arterial spasmolysis was performed using a standardized nimodipine dose per treated vascular territory according to institutional routine. The number of treated vessels/territories per session was individualized based on angiographic vasospasm distribution. Repeated endovascular treatments were performed when recurrent or persistent vasospasm was suspected clinically (new/worsening neurological deficit) and/or imaging-based (new/progressive perfusion deficit on CTP with Tmax > 6 s [18]), and angiographically confirmed on DSA.

### 2.3. Assessed Variables and Primary Outcome

Patients’ characteristics were retrospectively extracted from the electronic hospital records. Collected variables included demographic data (age, sex) and relevant clinical information, such as medical history, admission status, treatment modality, and in-hospital course.

Functional outcome was assessed using the modified Rankin Scale (mRS). The primary outcome of the study was in-hospital mortality, defined as an mRS score of 6 during hospitalization.

All patients underwent either computed tomography (CT) or magnetic resonance (MR) imaging following endovascular treatment or neurosurgical intervention, and at hospital discharge when available. Post-treatment and discharge imaging studies were independently reviewed by two board-certified neuroradiologists who were blinded to all clinical data and patient outcomes. Imaging was specifically evaluated for the presence of SVS-I. In cases of disagreement, consensus was achieved through joint review.

### 2.4. Statistical Analysis

All statistical analyses were performed using parametric and non-parametric methods based on data distribution. Continuous variables were first assessed for normality and homogeneity of variance using the Shapiro–Wilk test. Categorical variables are reported as absolute frequencies and proportions (percentages) and were compared between groups using the chi-square (χ^2^) test. Continuous variables with a normal distribution are presented as means with corresponding standard deviations (SD) and were analyzed using the unpaired Student’s t-test. Non-normally distributed continuous variables are reported as medians with interquartile ranges (IQR) and were compared using the Mann–Whitney U test.

A confounder-adjusted mediation analysis was performed using a regression-based path approach. The model estimated the indirect effect via SVS-I and the direct effect of treatment timing on mortality. Adjustments were made for age, sex, Fisher grade, vasospasm location, external ventricular drainage, hypertension, and smoking status.

Results of the multivariable logistic regression models were presented as odds ratios, with 95% confidence intervals and corresponding *p*-values. A *p*-value < 0.05 was defined as statistically significant. Statistical analyses were performed with Stata/MP, version 17.0 (StataCorp LLC, College Station, TX, USA).

## 3. Results

### 3.1. Study Population

**Figure 3:**
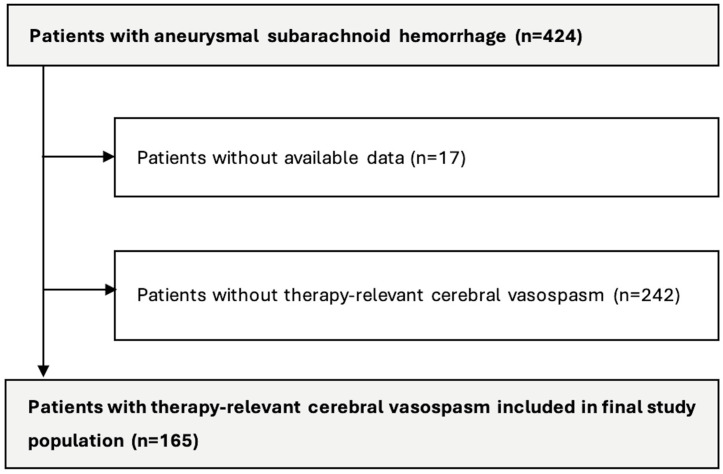
Flow chart of patient inclusion.

Overall, 424 patients with aneurysmal subarachnoid hemorrhage (aSAH) treated between January 2016 and May 2024 were screened. Of these, 165 patients developed treatment-relevant cerebral vasospasm (CV) following aSAH and fulfilled the inclusion criteria (Figure 3). All patients were treated with intra-arterial spasmolysis; mechanical dilatation was not performed in this patient cohort. The mean age of the cohort was 56.6 ± 11.7 years, and 111 patients (67.2%) were female. The mean body mass index was 26.6 ± 4.7 kg/m^2^. Arterial hypertension was the most common cardiovascular risk factor, present in 47.3% (n = 78) of patients, while 33.9% (n = 56) had a history of active smoking.

Aneurysms were predominantly located in the anterior circulation (75.8%), with the anterior cerebral artery being the most frequent site (46.1%), followed by the middle cerebral artery (13.9%) and internal carotid artery (9.1%). Posterior circulation aneurysms accounted for 24.2% of cases. No significant differences in aneurysm location were observed between patients who died in hospital and those discharged alive. EVD was required in 130/165 patients (78.8%).

With regard to imaging characteristics, the overall median modified Fisher grade was 3 (IQR 2–3). Patients who died in hospital had significantly higher hemorrhage burden, reflected by a median modified Fisher grade of 4 (IQR 4–4), compared with 3 (IQR 2–4) in survivors (*p* < 0.001). Intracerebral hemorrhage was present in 41.2% of the total cohort, without a statistically significant difference between outcome groups. Vasospasm-associated cerebral infarction occurred in nearly half of all patients (47.3%) but was markedly more frequent among patients who died in hospital (91% vs. 40.6%; *p* < 0.001).

Clinical severity on admission differed substantially between groups. The median Hunt and Hess grade in the overall cohort was 3 (IQR 2–4), but patients who died exhibited significantly higher grades (median 4 [IQR 3–5]) compared with survivors (median 2 [IQR 1–3]; *p* < 0.001). Notably, mortality was significantly higher among non-smokers than smokers (36% vs. 18%; *p* < 0.001).

Regarding treatment characteristics, 25.5% of patients underwent surgical clipping, while the remainder were treated endovascularly, with no significant differences between outcome groups. External ventricular drainage was required in 78.8% of patients, again without significant group differences. The mean time from aSAH onset to first intra-arterial spasmolysis was 8.1 ± 3.7 days. Patients who died in hospital underwent spasmolysis significantly earlier than survivors (6.2 ± 3.5 vs. 8.4 ± 3.7 days; *p* = 0.008). Repeated sessions of intra-arterial spasmolysis (≥2) were performed in 78/165 patients (47.3%).

A comprehensive overview of baseline characteristics, imaging findings, treatment details, and outcomes stratified by in-hospital mortality is provided in Table 1.

### 3.2. Primary Outcome

Overall, 13.3% of patients (n = 22) died during the hospital stay, with death occurring after a mean of 22.4 days (SD ± 3.4), representing the primary outcome of the study. Mortality decreased with the later onset of treatment-relevant CV. Stratified by early (≤5 days), intermediate (6–10 days), and late (≥11 days) CV onset, in-hospital mortality rates were 24%, 13%, and 3%, respectively.

### 3.3. Mediation Analysis

**Figure 4:**
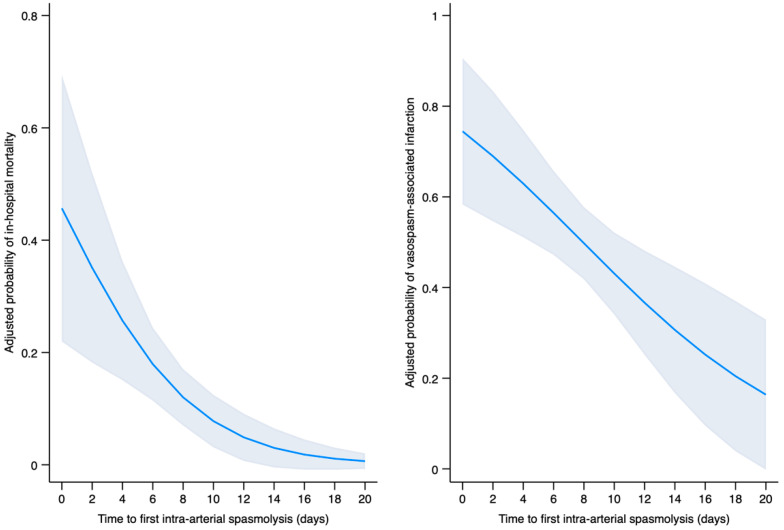
Adjusted predictive probability of in-hospital mortality and vasospasm-associated infarction in relation to time (in days) to first endovascular intra-arterial spasmolysis.

**Figure 5:**
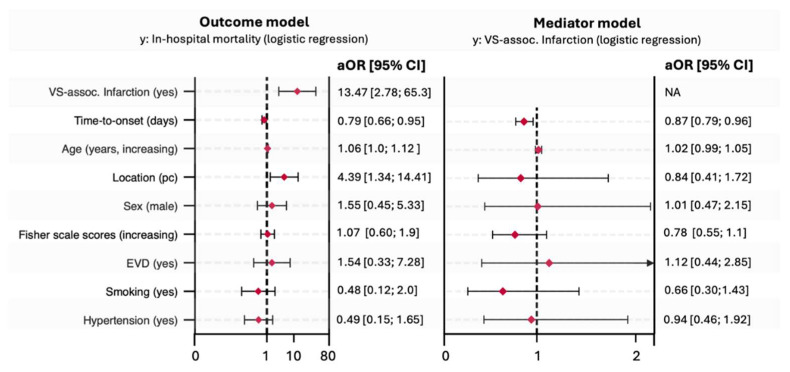
Adjusted logistic regression analyses of the outcome and mediator model. Legend: VS. assoc. Infarction = Vasospasm-associated infarction. EVD = External ventricular drainage. Pc = Posterior circulation. Smoking = Active smoker or history of smoking.

In multivariable logistic regression analysis, SVS-I (adjusted odds ratio [aOR] of 13.47 (95% CI: 2.78 to 65.3; Figure 4), earlier onset of CV necessitating intra-arterial spasmolysis (aOR: 0.79; 95% CI: 0.66 to 0.95; Figure 4), posterior circulation aneurysm location (aOR: 4.39; 95% CI: 1.34 to 14.41), and age (aOR: 1.06 per year increase; 95% CI: 1.01 to 1.12) were significantly associated with in-hospital mortality (Figure 5).

**Figure 6:**
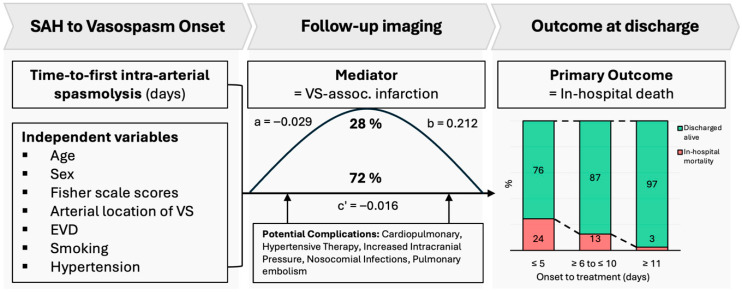
Mediation model layout and result overview. Legend: VS. assoc. Infarction = Vasospasm-associated infarction. EVD = External ventricular drainage. Arterial location = Reference Posterior circulation. Smoking = Active smoker or history of smoking.

All requirements for establishing a mediation relationship according to the criteria by Baron and Kenny were met [19]. The regression analysis of the direct path revealed a significant association between time-to-first intra-arterial spasmolysis and in-hospital mortality. A significant association was observed between SVS-I and both the independent variable and the outcome, confirming its role as a mediator. The total effect of earlier onset of CV on in-hospital mortality was −2.3 percentage points (pp) (95% CI: −4.3 to −0.3 pp, *p*  =  0.024), of which −0.6 pp (95% CI: −1.2 to −0.2 pp, *p*  =  0.019) was mediated by SVS-I (Table 2). This corresponds to a 28% proportion of the total effect being explained by SVS-I. The direct effect of time to onset of CV on in-hospital mortality remained significant at −1.6 pp (95% CI: −3.1 to −0.2 pp, *p*  =  0.024; Figure 5 and Figure 6).

## 4. Discussion

In this study examining the relationship between time from aSAH to the onset of cerebral vasospasms, SVS-I, and in-hospital mortality, we observed the following main findings: (1) A total of 13% of patients with CV following aSAH died during hospitalization. (2) Earlier onset of CVs necessitating intra-arterial spasmolysis was associated with an increased risk of in-hospital mortality. (3) Mediation analysis revealed that 28% of the effect of early onset of CV on in-hospital mortality was mediated by SVS-I. (4) Multivariable logistic regression analysis marked advanced age and posterior circulation aneurysms as further factors associated with in-hospital mortality.

In the literature, in-hospital mortality in patients with CV following aSAH ranges from 17% to 34% [20,21,22]. In our study, we observed slightly lower in-hospital mortality, with an overall rate of 13%. This difference might be explained by our study population, which focuses only on patients with aSAH and subsequent CV development who received i.a. spasmolysis. Patients with early severe brain injuries, such as herniation or parenchymal brain hemorrhage with mass effect, who died within the first days, were not included.

CVs typically occur within the first 14 days following aSAH, with the most frequent onset around day 7 [6,7,8]. An earlier onset than average may reflect a more pronounced response of the arterial vessel walls to irritation caused by subarachnoid hemorrhage, which may increase chances of further complications such as delayed cerebral ischemia and ultimately increased mortality. Furthermore, patients with an early onset of CV after aSAH are at high risk of developing secondary ischemic infarction [23,24,25]. Corroborating these previous findings, adjusted multivariable logistic regression analysis showed that both early CV onset requiring i.a. spasmolysis and the development of secondary infarction due to cerebral vasospasm were strongly associated with in-hospital mortality. This finding is in line with the study of Rabintein et al., reporting that the mortality rate was significantly higher in patients with SAH and cerebral infarction compared to those without cerebral infarction (23% vs. 5%) [26]. To further explore the relation between early onset of CV, secondary infarction, and in-hospital mortality, we conducted a confounder-adjusted mediation analysis, which revealed that 28% of the effect of early onset of CV on in-hospital mortality was mediated by SVS-I. This finding highlights the critical role of SVS-I as a partial pathophysiological mediator in the cascade linking early vasospasm to poor clinical outcomes. This association highlights the clinical impact of early screening and detection of CV within the first days after aSAH to identify subclinical or rapidly progressing vasospasm and prevent early complications and mortality. The remaining 72% of the effect of early CV on in-hospital mortality may be attributed to increased risk of systemic complications in the context of aSAH and CV, including cardiac and pulmonary disorders, disorders of sodium and water metabolism, hematological and immunological changes, and inflammatory responses [27,28].

Furthermore, we observed that advanced age was associated with higher odds for in-hospital mortality. This relation has been well investigated by multiple studies [25,29,30,31,32]. A nationwide cohort study in Norway, which analyzed discharge data of patients with aSAH over a six-year period, identified advanced age as a predictor of 30-day mortality in 1732 patients. The 30-day case fatality rate increased dramatically from approximately 27% in patients under 25 years old to around 61% in those over 85 years old. Similar to our results, this study observed, in addition to age, an association between posterior circulation aneurysms and mortality in multivariable analysis [33]. The proximity of the posterior circulation to the brainstem and the fourth ventricle, with a high chance for intraventricular hemorrhage and developing hydrocephalus, may explain the complications and fatal effects of this particular aneurysm site [34,35].

Prospective studies are needed to validate time-to-vasospasm onset as a prognostic marker and to integrate it with clinical, imaging, and biological parameters in robust risk prediction models. Leveraging advanced imaging, inflammatory biomarkers, and continuous neuromonitoring may facilitate earlier identification of high-risk patients. Randomized trials in patients with very early vasospasm are needed to determine whether early therapeutic escalation improves survival and functional outcomes.

This study has all the limitations that come with a single-center, retrospective design, including a limited sample size and limited generalizability of findings to other populations. Because there is no standardized definition of clinically relevant cerebral vasospasms, it is difficult to directly compare our findings with those of other studies that applied different diagnostic and treatment criteria for endovascular management of vasospasms. Furthermore, our institution’s algorithm for detecting cerebral vasospasms may differ from those of other institutions. ICP-related variables were not systematically available; therefore, EVD placement was used as a proxy for hydrocephalus severity in adjusted models.

## 5. Conclusions

Early onset of cerebral vasospasm is associated with an increased risk of in-hospital mortality following aneurysmal subarachnoid hemorrhage. This effect is partially mediated by secondary vasospasm-associated infarction. Patients with very early vasospasm onset may benefit from intensified early monitoring (TCD and early CTA/CTP when indicated) and timely interdisciplinary evaluation with treatment escalation to prevent infarction-related mortality. Further prospective multicenter studies are needed to externally validate our findings and assess their generalizability across different monitoring and treatment protocols.

## Figures and Tables

**Table 1:** Patients’ baseline characteristics compared by outcome groups.

Baseline Characteristics	All Patients (n = 165)	Death in Hospital (n = 22)	Discharged Alive (n = 143)	*p*-Value
■ Age (years), mean ± SD	56.6 (11.7)	59.3 (11.2)	56.2 (11.8)	0.249
■ Female sex, n (%)	111 (67.2)	15 (68)	96 (67.9)	0.88
■ BMI (kg/m^2^), mean ± SD	26.6 (4.7)	25.7 (4)	26.7 (4.7)	0.368
■ Hypertension, n (%)	78 (47.3)	9 (40.1)	69 (48.3)	0.284
■ Smoker, n (%)	56 (33.9)	4 (18.2)	52 (36.4)	<0.001
Aneurysm location, n (%)				
■ Anterior circulation				
- ICA	15 (9.1)	1 (4.5)	14 (9.8)	0.719
- MCA	23 (13.9)	3 (13.6)	20 (14)	0.2
- ACA	76 (46.1)	8 (36.4)	68 (47.6)	0.316
■ Posterior circulation	40 (24.2)	10 (45.6)	40 (28)	0.190
Imaging details				
■ Modified Fisher grade, median (IQR)	3 (2–3)	4 (4–4)	3 (2–4)	<0.001
■ ICH, n (%)	68 (41.2)	7 (31.8)	61 (42.7)	0.151
■ Vasospasm-associated infarction, n (%)	78 (47.3)	20 (91)	58 (40.6)	<0.001
Clinical deficit				
■ Hunt and Hess grade, median (IQR)	3 (2–4)	4 (3–5)	2 (1–3)	<0.001
Treatment details, n (%)				
■ Treatment (Clipping)	42 (25.5)	6 (27.3)	36 (25.1)	0.345
■ EVD	130 (78.8)	19 (86.4)	111 (77.6)	0.378
■ Days to first spasmolysis, mean ± SD	8.1 (3.7)	6.2 (3.5)	8.4 (3.7)	0.008
Clinical outcome				
■ mRS at follow-up, median (IQR)	1 (0–5)	6 (6–6)	0 (0–3)	<0.001

**Table 2:** Mediation analysis results.

	Outcome: In-Hospital Mortality Independent Variable: Time-to-First i.a. Spasmolysis Mediator: Vasospasm-Associated Infarction		
Increase in Probability of Outcome	Estimate	95% CI	*p*-Value
■ Total effect	−0.023	−0.043; −0.003	0.024
■ Direct effect	−0.016	−0.031; −0.002	0.024
■ Effect mediated by SVS-I	−0.006	−0.012; −0.002	0.019

## Data Availability

The data presented in this study are not publicly available due to legal and ethical restrictions related to patient privacy. Anonymized data may be available from the corresponding author upon reasonable request and with appropriate approvals.
